# Duodenal Biopsies for Coeliac Disease: Does Size Matter?

**DOI:** 10.3390/diagnostics15081000

**Published:** 2025-04-14

**Authors:** Mohamed G. Shiha, Francesca Manza, Suneil A. Raju, Andrew D. Hopper, Simon S. Cross, David S. Sanders

**Affiliations:** 1 Academic Unit of Gastroenterology, Sheffield Teaching Hospitals, Sheffield S10 2JF, UK; 2 Department of Translational Medicine, St. Anna Hospital, University of Ferrara, 44121 Ferrara, Italy

**Keywords:** coeliac disease, glutens, diagnosis, biopsy

## Abstract

**Background/Objectives**: Most adult patients require endoscopy and duodenal biopsies to diagnose coeliac disease. However, individuals who are unwilling or unable to undergo conventional endoscopy are left without diagnostic options or a formal diagnosis. We aimed to determine whether the small-sized biopsy forceps used during the more tolerable transnasal endoscopy (TNE) can provide adequate duodenal biopsy specimens for diagnosing coeliac disease. **Methods**: We prospectively recruited adult patients (≥18 years) with suspected coeliac disease between May and July 2024. All patients underwent peroral endoscopy, with four biopsies taken from the second part of the duodenum (D2) and one from the duodenal bulb (D1) using standard 2.8 mm biopsy forceps. The biopsy protocol was then repeated using smaller 2 mm biopsy forceps. Expert pathologists evaluated all samples for size, quality, and Marsh classification. **Results**: Ten patients (median age 45 years, 50% female) were included in this study, of whom seven (70%) were diagnosed with coeliac disease. In total, 100 duodenal biopsy specimens were collected and analysed (50 using standard biopsy forceps and 50 using smaller biopsy forceps). The size of D2 biopsies was significantly larger when using standard biopsy forceps compared with smaller forceps (4.5 mm vs. 3 mm, *p* = 0.001). Similarly, biopsies from D1 were also larger with standard forceps (3 mm vs. 2 mm, *p* = 0.002). Smaller forceps provided sufficient material for accurate classification in all cases, and the agreement between biopsies obtained using both forceps in D2 and D1 was 100% (k = 1.0). **Conclusions**: This pilot study demonstrates that small-sized biopsy forceps, used during TNE, can provide adequate tissue for histopathological diagnosis in patients with suspected coeliac disease. These findings pave the way for considering TNE as a more tolerable alternative to conventional endoscopy in diagnosing coeliac disease.

## 1. Introduction

Coeliac disease is a chronic autoimmune disorder triggered by the ingestion of gluten-containing food in genetically predisposed individuals [1]. It affects approximately 1% of the population worldwide and has had a rising incidence over the past two decades [2,3]. Yet, a substantial number of cases remain undiagnosed or face significant delays in diagnosis [4]. Delayed diagnosis is associated with a wide range of complications and has a substantial impact on patients’ physical and psychological well-being, as well as a higher impact on healthcare systems [5].

The current diagnostic algorithm for coeliac disease is a two-step approach that starts with serological testing for IgA tissue transglutaminase (IgA-tTG) and endomysial antibodies (EMAs) of patients with clinical suspicion of coeliac disease and first-grade relatives of patients with confirmed coeliac disease, followed by endoscopy and duodenal biopsies in those with positive serological results [6]. Biopsy specimens should contain at least three consecutive villous-crypt units visualized in their entirety and arranged parallel to each other. The most used histological classification of coeliac disease relies on the modified Marsh scale, which evaluates the number of intraepithelial lymphocytes, architectural features (such as villous atrophy and crypt hyperplasia), and the level of infiltration of the lamina propria. The final histological summary usually reports results based on the most severe lesions found among the biopsies performed [7,8].

Growing evidence suggests that in selected adult patients with very high titres of IgA-tTG (above 10 times the upper limit of normal [ULN]), a diagnosis of coeliac disease could be made without the need for histological confirmation [9]. While this approach is now a common clinical practice to diagnose children [10], current guidelines do not recommend this strategy for adults [8,11,12]. In addition, for most patients who have borderline or mildly raised serological markers, biopsies remain essential to confirm the diagnosis. One of the challenges in the current diagnostic pathway is related to patients who are unable or unwilling to undergo endoscopy due to its invasive nature, as they are left without alternative diagnostic options or a formal diagnosis.

Transnasal endoscopy (TNE) is a more tolerable alternative to conventional endoscopy. TNE uses an ultra-thin endoscope with a diameter < 6 mm that can be inserted via a nasal or oral route. The procedure is typically performed with the patient sitting upright, using a local nasopharyngeal anaesthesia, without the need for conscious sedation. The risks and discomfort of the procedure are therefore significantly lowered: complications related to the usage of benzodiazepine and opioids are avoided, and it has been shown to significantly lower the rate of cardiopulmonary events [13]. Moreover, it has been highlighted that TNE is significantly more comfortable than transoral endoscopy, with the most common issue being pain on insertion, whereas gagging is reduced [14,15]. Other benefits include a shorter procedure time and lower costs related to a lower rate of procedural failings and minimization of the observation time required after the procedure [13]. TNE has been widely studied in Barrett’s oesophagus surveillance, oesophageal varices screening, and the early detection of gastric cancer. However, currently available TNE endoscopes have a working channel with an approximately 2–2.4 mm diameter versus a 2.8 mm working channel in a standard endoscope [16], allowing the passage of smaller accessories only. Therefore, TNE is not currently recommended for patients with suspected coeliac disease: the smaller biopsy forceps used during TNE procedures have not been sufficiently evaluated for their ability to obtain adequate tissue samples for accurate histopathological diagnosis.

The aim of this pilot study was to determine whether small-sized biopsy forceps used during TNE can provide adequate duodenal biopsy specimens for diagnosing coeliac disease. For this purpose, we compared, in every patient, the histological Marsh classification of duodenal specimens obtained with standard forceps and duodenal specimens obtained with smaller forceps suitable for TNE.

## 2. Materials and Methods

### 2.1. Study Design and Participants

This prospective study was undertaken at the NHS England National Centre for Refractory Coeliac Disease at Sheffield Teaching Hospitals, United Kingdom, between May and July 2024. Adult patients (≥18 years) with suspected coeliac disease (an IgA-tTG titre greater than 7 U/mL) who had no contraindications to endoscopy were eligible for inclusion. All patients provided written consent to be included in this study. We collected basic demographic data, clinical data, and laboratory findings, including coeliac serology results and clinical phenotypes. All patients underwent standard oesophago-gastroduodenoscopy (OGD) and had their duodenal biopsies taken with standard biopsy forceps first (2.8 mm) and with TNE biopsy forceps (2 mm) afterwards, via the same gastroscope.

### 2.2. Sample Size

Our sample size was based on a previous pilot study, conducted by Saeian et al., which compared two biopsy forceps (2.2 mm vs. 1.8 mm) in the assessment of Barrett’s oesophagus [17]. That study included 10 patients, with quadrantic biopsy specimens obtained by each size of biopsy forceps at different levels for a total of 80 biopsies. Therefore, we considered a similar sample size of 10 patients (100 biopsy specimens) sufficient to provide preliminary data on the feasibility and diagnostic performance of these two sizes of biopsy forceps.

### 2.3. Endoscopy and Biopsy Protocol

All procedures were performed trans-orally by two accredited endoscopists using high-definition gastroscopes (Olympus GIF-H290 and GIF-EZ1500, Olympus Corporation, Tokyo, Japan). Conscious sedation with intravenous midazolam and fentanyl was used during all procedures. The duodenal mucosal macroscopic appearance was classified as either normal or suggestive of villous atrophy.

Four biopsies were taken from the second part of the duodenum (D2) and one from the duodenal bulb (D1) using the single-bite technique with standard 2.8 mm biopsy forceps (Radial Jaw™ 4, Boston Scientific, Marlborough, MA, USA). Afterwards, smaller 2 mm biopsy forceps (EndoJaw™, Olympus Corporation, Tokyo, Japan) were inserted via the same endoscope and the entire protocol was repeated for comparison. From each patient, we collected two D1 specimens (one with 2.8 mm forceps and one with 2 mm forceps) and eight D2 specimens (four with 2.8 mm forceps and four with 2 mm forceps).

### 2.4. Histopathological Assessment

Duodenal mucosal biopsy specimens were immediately placed in 10% formalin solution in the endoscopy room. All samples were then oriented by experienced biomedical scientists in the histopathology laboratory, sliced into sections, and stained using hematoxylin and eosin (H&E). For each patient, biopsies were separated into four pots: two separate pots for standard forceps biopsies, one containing the D1 sample and one containing four D2 samples, and two for smaller forceps biopsies (one containing the D1 sample and one containing four D2 samples). All specimens were examined by two experienced gastrointestinal pathologists and graded according to the Marsh classification [18]. Marsh 3 lesions were considered diagnostic for coeliac disease.

Biopsy samples were compared by size, tissue quality, and Marsh classification to assess differences between standard and smaller forceps. Blinding was not feasible due to the clear size difference between specimens obtained by standard and smaller forceps.

### 2.5. Ethical Approval

This study was approved by Yorkshire and the Humber Research Ethics Committee (24/YH/0016) on the 27 February 2024, IRAS ID 331944, and prospectively registered on the NIHR open database (CPMS ID 60536).

### 2.6. Statistical Analysis

Descriptive statistics were used to summarise the baseline characteristics of patients, including demographic data, clinical findings, and biopsy results. Continuous variables were reported as medians with interquartile ranges, while categorical variables were presented as frequencies and percentages. The sizes of biopsies using standard and small biopsy forceps were compared using the Mann–Whitney U test. The level of agreement between these two biopsy techniques (standard and small forceps) was assessed using Cohen’s kappa coefficient (κ) to determine the consistency in Marsh classification grading between the two methods. A kappa value of 0.61–0.80 was considered substantial agreement, and values above 0.81 indicated almost perfect agreement. All statistical analyses were performed using Stata version 18 (StataCorp LLC, College Station, TX, USA).

## 3. Results

### 3.1. Participant Characteristics

A total of 10 adult patients (median age 45 years, 50% female) were included in this study, of whom 7 (70%) were diagnosed with coeliac disease. Half of these patients presented with gastrointestinal symptoms, including abdominal pain, bloating, and diarrhoea. Detailed demographic and clinical characteristics of these participants are summarised in Table 1.

### 3.2. Endoscopy and Biopsies

No adverse events were reported during biopsy procedures, and all patients tolerated the endoscopy without complications. Four biopsies were taken from the second part of the duodenum (D2) and one from the duodenal bulb using the single-bite technique with standard 2.8 mm biopsy forceps (Radial Jaw™ 4, Boston Scientific). Next, four biopsies were taken from D2 and one from the duodenal bulb using the smaller 2 mm biopsy forceps (EndoJaw™, Olympus) for comparison.

### 3.3. Histopathological Assessment and Comparison of Biopsy Forceps Samples

In total, 100 duodenal biopsy specimens were collected and analysed (50 using standard biopsy forceps and 50 using the smaller biopsy forceps). The size of D2 biopsies was significantly larger when using standard biopsy forceps compared with the smaller forceps (4.5 mm vs. 3 mm, *p* = 0.001). Similarly, biopsies from D1 were also larger when taken with standard forceps (3 mm vs. 2 mm, *p* = 0.002).

Histopathological assessment using the Marsh classification showed no difference in classification between standard and small forceps. Smaller forceps provided sufficient material for accurate classification in all cases, and the agreement between biopsies obtained using both forceps in D2 and D1 was 100% (k = 1.0), as shown in Table 2.

**Figure 1:**
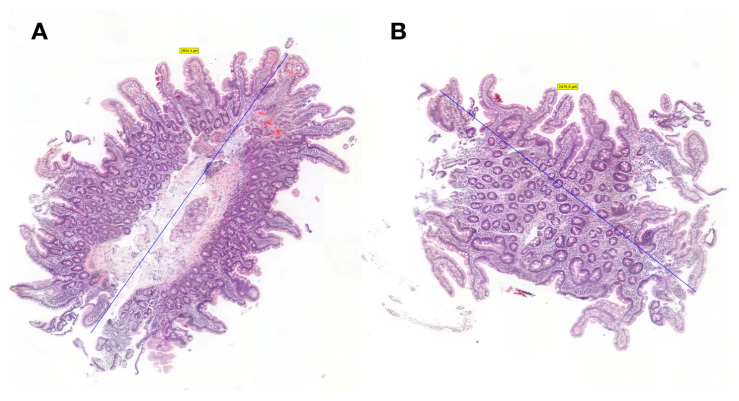
Comparison of duodenal biopsies obtained using standard and small forceps. (**A**) Standard duodenal biopsy showing normal duodenal mucosa; the biopsy was 2.8 mm in maximum dimension, as shown by the blue line. (**B**) Small size duodenal biopsy from the same patient showing normal duodenal mucosa; the biopsy was 2.5 mm in maximum dimension, as shown by the blue line.

Representative duodenal biopsy specimens obtained with small forceps compared with standard forceps are illustrated in Figure 1.

## 4. Discussion

To our knowledge, this is the first study to compare the use of small-sized and standard biopsy forceps for obtaining duodenal biopsies in patients with suspected coeliac disease. In this pilot study, we analysed 100 biopsies from 10 patients (50 using standard forceps and 50 using small forceps). Our results showed that while using standard forceps resulted in significantly larger biopsy specimens, small forceps provided quantitatively and qualitatively adequate samples for histopathological evaluation, with 100% agreement in Marsh classification between biopsies obtained using these two different types of forceps.

There is growing evidence supporting the use of TNE as a replacement for standard OGD. A recent study demonstrated how TNE outperformed standard OGD in some key procedural aspects, such as retroflexion and duodenal intubation [19]. TNE has shown higher-quality images in the oesophagus and comparable quality and definition in the stomach and duodenum. More importantly, TNE had a higher success rate in complex cases, allowing the completion of the procedure where standard OGD failed. In addition, TNE has been shown to be more cost-effective, reducing the workforce needed, the duration of procedures, and the carbon footprint, ultimately relieving pressure on busy endoscopy services [19,20]. However, the factor that probably decreased the penetrance of TNE in common practice is mainly the lack of evidence supporting the non-inferior quality of bioptic specimens.

Our findings are in line with previous studies that evaluated the use of small-sized biopsy forceps through ultra-thin transnasal endoscopy (TNE) in patients with different gastrointestinal conditions [17,21,22,23], which showed that despite the smaller size of specimens using small biopsy forceps, there were no differences in histological diagnoses compared with standard-sized forceps. Although these studies mainly focused on conditions affecting the oesophagus and stomach, some of them reported details concerning duodenal specimens. Al-Karawi et al. [22] performed duodenal biopsies with 2 mm forceps in three cases (in addition to gastric sampling), and in all cases the samples were reported as adequate for histopathologic assessment. Walter et al. [23] analyzed a total of 1335 histological pieces, of which 207 were duodenal biopsies sampled with TNE or standard OGD (with and without sedation). The authors concluded that even though the specimens obtained with TNE were smaller, their thickness was comparable with those collected with standard OGD; therefore, diagnostic performance was not significantly affected. These results are consistent with the outcomes highlighted in our study. However, previous studies were mainly focused on assessing oesophageal and gastric conditions, rather than specific to coeliac disease, and they included a relatively small number of duodenal biopsies.

The diagnosis of coeliac disease ultimately relies on compatible histological findings. In many cases, interpretation of duodenal biopsies in patients with suspected coeliac disease can be particularly challenging, and adequate tissue samples are crucial for identifying subtle mucosal and villous changes characteristic of coeliac disease [24]. Therefore, obtaining high-quality, well-oriented duodenal biopsies with a sufficient amount of tissue is essential to accurately assess intraepithelial lymphocytosis, crypt hyperplasia, and villous atrophy, the key components of the Marsh classification [18]. Conversely, poor-quality biopsies can lead to misclassification, missed diagnoses, and conflicting results, ultimately increasing the pressure and expenses on healthcare systems and, more importantly, prolonging patients’ diagnostic delay and lowering the standard of care.

Our pilot study provides reassuring results that the small biopsy forceps used in the more tolerable TNE can deliver sufficient diagnostic tissue for patients with suspected coeliac disease. We showed that all 50 biopsies taken with TNE-suitable forceps provided enough material to assess specimens according to Marsh classification. These results could pave the way for offering TNE to patients with suspected coeliac disease as a more tolerable alternative to conventional endoscopy. In addition, TNE could represent an acceptable solution for patients who cannot undergo sedation and a cheaper option for healthcare systems [17,20]. Notably, amongst autoimmune disorders, coeliac disease has shown the largest increase in incidence in the UK between 2000 and 2019, and it is expected to grow further [25]. Therefore, alternative solutions that are more cost- and time-efficient are highly needed to relieve the pressure on healthcare systems. Yet, larger prospective studies using TNE are needed to evaluate the accuracy and tolerability of TNE in this specific cohort of patients with coeliac disease.

The key strength of this study was the direct comparison between small-sized and standard biopsy forceps: all biopsy samples were collected in the same condition, allowing a proper evaluation specifically targeting biopsy instruments and samples. In addition, all patients included in this study belonged to a well-defined patient group with positive IgA-tTG titres, and the same standardised biopsy protocol was used for both bioptic techniques. Moreover, our results provide novel data that may have a direct impact on clinical practice by establishing an evidence base for a cheaper and better-tolerated diagnostic option specifically for patients with suspected coeliac disease.

Our study also had some limitations. First of all, it was conducted at a single centre of excellence, with high levels of expertise in both endoscopy and coeliac disease, which may limit the generalisability of our findings to other clinical settings and smaller realities. Second, our study involved a limited number of patients, as this was a pilot study, lowering the generalisability of our results. Therefore, larger cohorts and multicentric studies are needed to validate these results. Third, the comparison between samples obtained by two sizes of biopsy forceps was undertaken by expert pathologists, which may not reflect real-world variability in clinical practice. Finally, although all samples were examined by two experienced gastrointestinal pathologists, both standard and small-sized biopsy specimens from the same patient were evaluated by one pathologist, which could have introduced confirmation bias. However, given the pathologists’ extensive experience, it is unlikely that this potential bias significantly influenced the overall results.

## 5. Conclusions

In conclusion, this pilot study demonstrated that small-sized biopsy forceps are effective for obtaining diagnostically adequate duodenal biopsies in patients with suspected coeliac disease, with no differences in terms of histopathological reports compared to standard biopsy forceps. These findings support the potential use of smaller forceps used in TNE procedures in diagnosing coeliac disease, offering patients more tolerable diagnostic options without compromising diagnostic accuracy.

## Figures and Tables

**Table 1:** Patient characteristics.

**Sex, *n* (%)**	
Female	5 (50)
Male	5 (50)
**Age in years, median (IQR)**	**45 (28–60)**
Presentation, *n* (%)	
Gastrointestinal symptoms	5 (50)
Extra-intestinal symptoms	2 (20)
Family history	4 (40)
Autoimmune disease screening	1 (10)
**Serology**	
tTG titre (U/mL), median (IQR)	17 (12.5–64.0)
EMA positivity *, *n* (%)	6/8 (75)
**Blood results, *n* (%)**	
Anaemia	0
B12 deficiency	0
Folate deficiency	3 (30)
Vitamin D deficiency	0
Calcium deficiency	1 (10)
**Endoscopy and histology ⱡ, *n* (%)**	
Macroscopic signs of atrophy	3 (30)
Marsh 0	3 (30)
Marsh 1	0
Marsh 2	0
Marsh 3	7 (70)

tTG, tissue transglutaminase; EMA, endomysial antibodies. * Two patients did not have EMA. ⱡ Histology results based on most severe lesions on standard biopsies.

**Table 2:** Comparison of Marsh classification in duodenal biopsies (D2 and D1) using standard and small biopsy forceps for each patient.

	Standard Biopsy Forceps		Small Biopsy Forceps	
	D2 Biopsies	D1 Biopsies	D2 Biopsies	D1 Biopsies
Patient 1	Marsh 0	Marsh 0	Marsh 0	Marsh 0
Patient 2	Marsh 3	Marsh 3	Marsh 3	Marsh 3
Patient 3	Marsh 1	Marsh 3	Marsh 1	Marsh 3
Patient 4	Marsh 3	Marsh 3	Marsh 3	Marsh 3
Patient 5	Marsh 3	Marsh 3	Marsh 3	Marsh 3
Patient 6	Marsh 3	Marsh 3	Marsh 3	Marsh 3
Patient 7	Marsh 0	Marsh 0	Marsh 0	Marsh 0
Patient 8	Marsh 3	Marsh 3	Marsh 3	Marsh 3
Patient 9	Marsh 3	Marsh 3	Marsh 3	Marsh 3
Patient 10	Marsh 0	Marsh 0	Marsh 0	Marsh 0

## Data Availability

Data are available upon reasonable request.

## Abbreviations

The following abbreviations are used in this manuscript:

TNE	Transnasal endoscopy;
D1	Duodenal bulb;
D2	Second part of the duodenum;
H&E	Hematoxylin and eosin;
K	Cohen’s kappa.
